# Transformation of myelodysplastic syndrome to mixed phenotype acute leukaemia

**DOI:** 10.1002/jha2.59

**Published:** 2020-07-19

**Authors:** Louis Do, John Giannoutsos

**Affiliations:** ^1^ Nepean Hospital Department of Haematology Derby St Kingswood NSW 2747 Australia


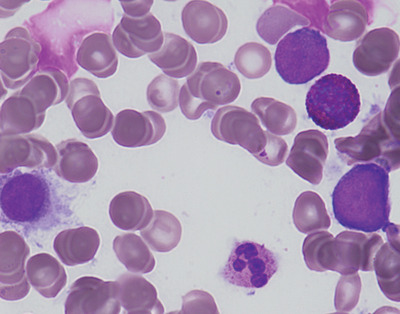



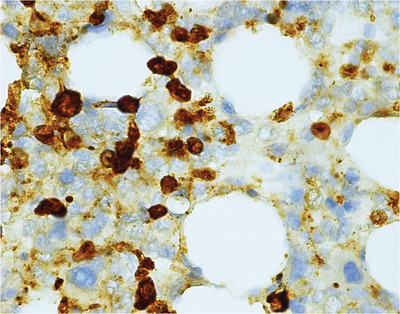



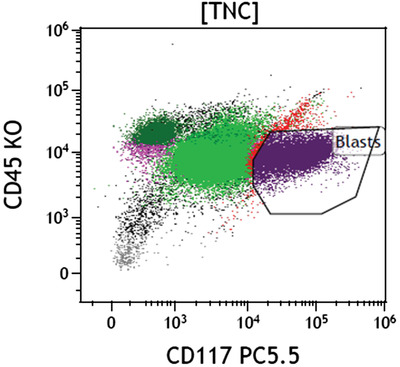


A 71‐year‐old man with de novo myelodysplastic syndrome with multilineage dysplasia and normal cytogenetics present with worsening cytopenias and was diagnosed with myelodysplastic syndrome with excess blasts‐2 (MDS‐EB2). He achieved good haematologic response following 12 cycles of azacitidine before cytopenias recurred. A bone marrow biopsy showed 25% blasts and background marked trilineage dysplasia.


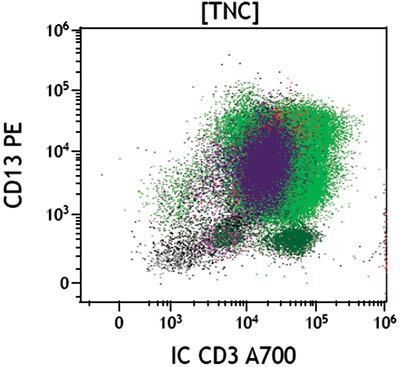



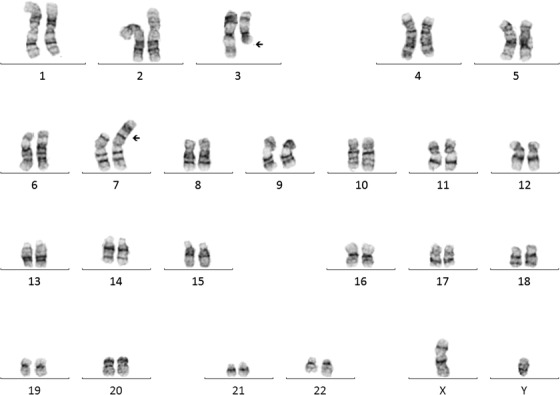


Blasts were large‐sized, with basophilic, agranular cytoplasm with prominent nucleoli. On trephine biopsy immunohistochemistry, the immature myeloid precursors express CD117 and a subset of these express myeloperoxidase. G‐banded karyotype showed a novel translocation between 3q and 7p in eight of 20 cells.

On flow cytometry, blasts comprise 19% of total nucleated cells, expressing CD45 weak+, CD117+, C34−, CD33−, CD38+, interestingly also CD7+, and cytoplasmic CD3 weak+.  The blasts therefore demonstrated features of both myeloid (CD117, MPO) and T‐lymphoid (cytoplasmic CD3) lineages, consistent with mixed phenotype acute leukaemia (MPAL), which had most likely transformed from MDS‐EB2.

MDS is considered primarily a myeloid malignancy, tending to transform to acute myeloid leukaemia. Transformation to MPAL or acute lymphoblastic leukaemia is much less commonly reported. The current case demonstrates transformation of de novo MDS to MPAL, which was supported by the presence of myeloid‐ and T‐lymphoid‐defining markers on immunohistochemical and flow cytometric methods.

